# Simplified Mechanistic Aging Model for Lithium Ion Batteries in Large-Scale Applications

**DOI:** 10.3390/ma18061342

**Published:** 2025-03-18

**Authors:** Zhe Lv, Huinan Si, Zhe Yang, Jiawen Cui, Zhichao He, Lei Wang, Zhe Li, Jianbo Zhang

**Affiliations:** 1 Beijing HyperStrong Technology Co., Ltd., Building 2C, No.9 Fenghao East Road, Haidian District, Beijing 100094, China; lvzhe@hyperstrong.com.cn (Z.L.); yangzhe@hyperstrong.com.cn (Z.Y.); cuijiawen@hyperstrong.com.cn (J.C.); hezhichao@hyperstrong.com.cn (Z.H.); wanglei@hyperstrong.com.cn (L.W.); 2 School of Vehicle and Mobility, Tsinghua University, Beijing 100084, China; zhe_li@tsinghua.edu.cn; 3 Institute of Artificial Intelligence in Sports, Capital University of Physical Education and Sports, Beijing 100191, China; sihuinan@cupes.edu.cn

**Keywords:** lithium ion battery, aging, mechanistic model, model simplification, large-scale application

## Abstract

Energy storage systems play a vital role in balancing solar- and wind-generated power. However, the uncertainty of their lifespan is a key factor limiting their large-scale applications. While currently reported battery aging models, empirical or semi-empirical, are capable of accurately assessing battery decay under specific operating conditions, they cannot reliably predict the battery lifespan beyond the measured data. Moreover, these models generally require a tedious procedure to determine model parameters, reducing their value for onsite applications. This paper, based on Newman’s pseudo-2D performance model and incorporating microparameters obtained from cell disassembly, developed a mechanistic model accounting for three major aging mechanisms of lithium iron phosphate/graphite cells, i.e., solid electrolyte interphase growth, lithium plating, and gas generation. The prediction of this mechanistic model agrees with the experimental results within an average error of ±1%. The mechanistic model was further simplified into an engineering model consisting of only two core parameters, loss of active lithium and loss of active material, and was more suitable for large-scale applications. The accuracy of the engineering model was validated in a 100 MW/200 MWh energy storage project. When the actual State of Health (SOH) of the battery degraded to 89.78%, the simplified model exhibited an error of −0.17%, and the computation time decreased from 8.12 h to 10 s compared to the mechanistic model.

## 1. Introduction

With the rapid development of the global economy, carbon emissions continue to rise, making renewable energy generation methods such as wind and solar power increasingly prioritized by countries worldwide. However, due to the inherent intermittency and unpredictability of wind and solar power, their large-scale integration into the power grid inevitably exerts substantial impacts, leading to a continuous decline in system rotational inertia and increasing the difficulty of power balancing. This has sharply raised the demand for energy storage systems. Nevertheless, the initial investment costs for large-scale electrochemical energy storage systems are high, and under current return-on-investment models, cost recovery often takes several years. Therefore, to effectively enhance the reliability of energy storage asset valuation, mitigate investment risks, and avoid the rapid degradation of storage system lifespan and potential safety issues, it is crucial to diagnose and optimize the health status of large-scale energy storage systems and establish predictive models to advance the energy storage industry.

However, due to the complex internal microstructure of storage battery cells and various interfacial reactions, accurately assessing battery lifespan presents significant challenges. Researchers have undertaken comprehensive investigations into the degradation mechanisms of the lithium ion battery lifespan, examining the phenomenon from both microscopic and macroscopic vantage points. These studies aim to precisely diagnose the SOH within energy storage systems and refine optimization strategies for enhanced performance and longevity.

During the usage of lithium-ion batteries, lifespan degradation can be divided into two categories: calendar life and cycle life. In terms of calendar life, Dubarry et al. [1] reviewed nearly 20 years of research on the calendar life of lithium ion batteries and compiled a comprehensive summary of the factors influencing the calendar life of batteries made from different materials. The findings indicate that the calendar life of almost all types of materials is closely related to the storage temperature and state of charge (SOC). At high SOC levels, the electrode surface potential is elevated, which accelerates side reactions with the electrolyte. High temperatures exacerbate irreversible side reactions, consuming active materials and active lithium within the battery, thus causing a loss of battery capacity.

For cycle life, the main influencing factors include cycling conditions, depth of discharge (DOD), and ambient temperature. The primary mechanisms affecting battery lifespan degradation focus on the loss of active material (LAM) in the electrodes and the loss of active lithium (LLI) [2]. Based on the aforementioned studies, researchers have developed various battery aging models. These models are generally categorized into purely theoretical mechanism-based aging models and empirical aging models. Mechanism-based degradation models start from the micro-mechanisms of battery degradation and use various micro-level parameters to accurately characterize the battery state [3,4]. Empirical models, on the other hand, are constructed using actual cell test data, featuring fewer parameters and a relatively simpler structure [5,6].

Su et al. [7] investigated the path dependence of the calendar life of ternary batteries under static and non-static conditions, and developed corresponding lifespan models. Through experiments, they compared calendar life degradation at 45 °C, 53 °C, and 60 °C across four SOC levels (40%, 60%, 80%, and 95%) under static storage and different storage paths. The study concluded that battery capacity degradation was independent of temperature and the SOC path, while the increase in internal resistance showed path dependence on SOC but was independent of the temperature path. Liu et al. [8] proposed a physics-based battery remaining useful life (RUL) prediction method. Unlike traditional empirical models, this method considers three main battery degradation mechanisms: loss of active material in the positive electrode (LAMPE), loss of active material in the negative electrode (LAMNE), and loss of active lithium. Using the test results of voltage and capacity, the model calibrates degradation parameters with a half-cell model, and then fits the degradation parameters using empirical models and the least squares method. Compared to traditional capacity models, this method demonstrated higher accuracy in RUL prediction, particularly in the early stages of battery lifespan degradation, where the precision difference was more pronounced.

At present, research on the aging mechanism and modeling still faces two challenges: at the research level, (1) the time-varying characteristics of capacity attenuation have not yet established the evolution law framework under multi-stress coupling conditions [9]; (2) the aging gradient distribution characteristics caused by multi-scale spatial heterogeneity of electrodes, cells, and modules need to be characterized by a three-dimensional characterization system [10,11]. Especially for hundreds of Ah commercial batteries, the phenomenon of non-uniform aging distribution caused by electrochemical–mechanical stress coupling has not formed a systematic research paradigm. In terms of modeling, (1) the existing mechanical–electrochemical coupling models are mostly limited to unipolar reaction path modeling such as the growth of the SEI film on the anode side [12,13], and lack the full-electrode co-evolution characterization of degradation mechanisms such as the phase transition of cathode-active materials. (2) It is difficult for the traditional model to effectively describe the aging distribution evolution law driven by multiple physical fields coupled with the temperature field, stress field, and concentration field under actual working conditions.

To address the aforementioned issues, this study innovatively introduced LAM and LLI attenuation mechanisms into the mechanism model, and successfully built a hundreds-of-Ah-energy storage battery life attenuation model, and based on the model simplification strategy, it has been fully applied and verified in the MWh-level energy storage power station. The systematic designs and experiments can be classified as the following three main aspects: constructing a mechanistic aging model for lithium iron phosphate/graphite (LFP/Graphite) batteries; simulating and verifying the results of the mechanistic aging model; and simplifying the mechanistic aging model to develop an engineering model. This provides theoretical support for optimizing lifespan control strategies.

In constructing the aging model for batteries, corresponding side reactions were incorporated into the typical mechanism model based on the identified degradation mechanisms. The cells were disassembled to obtain micro-level parameters of the battery. Additionally, using laboratory data from aging calibration experiments, a mechanistic aging model was developed. Through iterative simulation calculations, transient solutions for batteries with different cycle counts were performed, allowing for the determination of LAM and LLI within the battery.

Furthermore, by simulating the mechanism models under various operating conditions, the variation patterns of LAM and LLI can be obtained. Using data fitting methods, the functional forms dominated by different mechanisms were identified. These were then applied to simplify the mechanism model based on the matching relationship between the anode and the cathode, enabling the estimation of the battery’s state of health (SOH) and the development of an engineering model. Subsequently, the calculation results of the engineering model under complex conditions were compared with experimental data to validate the model’s accuracy. A sensitivity analysis of the factors affecting battery lifespan was conducted, and the model was ultimately applied to diagnose the health status of energy storage systems and optimize control strategies.

## 2. Construction of Mechanistic Model

Side reactions occurring within the battery are a major cause of aging, and different operating conditions can lead to different types of side reactions. A battery mechanistic model was built in this section with three different side reactions such as the solid electrolyte interphase (SEI) growth, lithium plating, and gas generation.

### 2.1. Impact of Three Major Side Reactions on Aging

Common side reactions include the growth and decomposition of SEI, lithium plating and dendrite growth, electrode particle fracture, graphite exfoliation, and the dissolution of transition metals [14,15]. Table 1 summarizes six side reaction mechanisms within the battery, and outlines the influence of various factors such as temperature, voltage, SOC, and current magnitude on each mechanism.

This study focused on the LFP/graphite system, which, compared to the more complex aging mechanisms of ternary systems, exhibits relatively simpler aging mechanisms, primarily involving three types [16,17,18]: SEI film growth, lithium plating, and gas generation.

#### 2.1.1. SEI Film Growth

The SEI film is a passivation layer on the surface of the anode, mainly consisting of an inner inorganic layer and an outer organic layer, and possesses the properties of a solid-state electrolyte. It forms during the initial cycles of the battery. The primary reason for its formation is that the stability window of the electrolyte is typically around 1–4.5 V (vs. Li/Li+), while the electrode potential of the graphite anode during extensive lithium intercalation is outside this stability window. This causes reactions between the electrode, the organic electrolyte, and lithium salts, resulting in the loss of available lithium. The SEI film acts as a barrier to prevent direct contact between the electrolyte and the electrode material surface, thereby avoiding further side reactions. However, due to the diffusion of solvent molecules, the fracture of particles or the SEI film, and the deposition of side reaction products, the electrode surface can become exposed to the electrolyte. Consequently, the growth of the SEI film continues throughout the battery’s lifecycle.

With the continuous growth of the SEI film, on the one hand, the impedance of the electrode particle surface will increase, and on the other hand, the porosity of the electrode will decrease. The formation of gas will occupy the pores of the electrode and cause some of the active particles to be deactivated. Under the leadership of the above two mechanisms, the battery will age, which will hinder the ion transport process, trigger a large concentration gradient, increase the risk of lithium deposition, and further accelerate SOH decay. At the same time, the porosity will change with the aging process, the loss of lithium ions will also cause the stoichiometric coefficient to appear as the “drift” phenomenon, and ultimately have an adverse impact on the entire process of electrochemical reaction from the external characteristics of the battery capacity decay and power decline.

The SEI film is composed of organic and inorganic components. In the mechanism model of this chapter, the reduction of the ethylene carbonate (EC) solvent on the anode surface was considered, without distinguishing the generated products.

(1) $$2C_{2}H_{4}CO_{3}+2e^{-}+2{Li}^{+}\to {\left(CH_{2}OCH_{2}Li\right)}_{2}↓+C_{2}H_{4}↑$$

The current density of the SEI film growth side reaction is primarily determined by the rate at which the EC solvent diffuses to the anode surface and the reaction kinetics, and it is calculated using the anodic Tafel equation.

(2) $$j_{SEI}=-k_{0,SEI}Fc_{EC}^{s}exp(-\frac{\alpha_{c,SEI}F}{RT}\eta_{2})$$

where ${\;j}_{SEI}\;$ represents the current density of the SEI film side reaction, $k_{0,SEI\;}$ is the reaction rate constant, F  is the Faraday constant, $c_{EC}^{s}\;$ is the concentration of EC that has diffused to the anode surface, $\alpha_{c,SEI}\;$ is the transfer coefficient (with a value of 0.5), and ${\;\eta }_{2}\;$ is the overpotential of the SEI film side reaction.

(3) $$\eta_{2}=\phi_{s}-\phi_{l}-j_{tot}R_{film}-U_{SEI}$$

$\phi_{s}$ is the solid-phase electrode potential, $\phi_{l}\;$ is the liquid-phase electrode potential, $j_{tot}\;$ is the total interfacial current density, $R_{film}\;$ is the resistance of the SEI film, and $\;U_{SEI}\;$ is the equilibrium potential of the side reaction, which is considered to be 0 V (vs. Li/Li+).

The EC solvent diffuses through the SEI film to the anode surface, where it undergoes reduction.

(4) $$-D_{EC}\frac{c_{EC}^{s}-c_{EC}^{0}}{\delta_{film}}=-\frac{j_{SEI}}{F}$$

$D_{EC\;}$ represents the diffusion rate of the EC solvent, $c_{EC}^{0}\;$ is the concentration of the EC solvent in the bulk electrolyte (a constant value), and ${\;\delta }_{film\;}$ is the thickness of the SEI film.

The change in SEI film thickness over time and the resulting impedance variation can be expressed by the following equation:

(5) $$\frac{\partial \delta_{film}}{\partial t}=\frac{j_{SEI}M_{SEI}}{2F\rho_{SEI}}$$

(6) $$R_{film}=\frac{\delta_{film}}{\kappa_{film}}$$

$M_{SEI}\;$ is the molar mass of the SEI film, $\rho_{SEI}\;$ is the molar density of the SEI film, t  is time, and ${\;\kappa }_{film}\;$ is the ionic conductivity of the SEI film.

As the SEI film grows, it gradually occupies the space in the anode’s pores, leading to a decrease in the anode porosity ($\varepsilon_{l}^{neg}$), where a is the proportionality coefficient.

(7) $$\frac{\partial \varepsilon_{l}^{neg}}{\partial t}=-a\frac{\partial \delta_{film}}{\partial t}$$

#### 2.1.2. Lithium Plating

Lithium plating refers to the process during charging when lithium ions fail to intercalate into the anode particles and instead receive electrons on the anode surface, forming metallic lithium. Lithium plating can be categorized into thermodynamic and kinetic plating. Thermodynamic plating occurs when the anode is fully lithiated, leaving no space for lithium to intercalate. Kinetic plating happens during fast charging, whereby the high potential of the electrolyte increases the rate of the side reaction compared to the main intercalation reaction. Lithium plating can occur even at 20 °C or 25 °C, primarily due to the potential for lithium plating. When the overpotential at the solid–liquid interface drops below 0 V (vs. Li/Li+) due to the increase in surface film resistance, lithium plating is likely to occur.

Lithium plating poses significant hazards to the battery. On one hand, the plated lithium reacts with the solvent to form a new SEI film. The growth of the SEI film causes lithium that has not reacted with the solvent to form “dead lithium”, reducing the amount of recyclable lithium and leading to rapid capacity decay. Additionally, pore blockage can result in decreased conductivity. On the other hand, severe lithium plating can lead to the growth of dendrites, which may pierce the separator and cause an internal short circuit.

The electrochemical equation for lithium deposition is as follows:

(8) $${Li}^{+}+e^{-}\to Li\left(s\right)↓$$

Lithium plating reaction current density is represented as follows:

(9) $$j_{lpl}=-j_{0,lpl}\exp\left(\frac{-\alpha_{c,lpl}F}{RT}\eta_{lpl}\right)$$

(10) $$\eta_{lpl}=\emptyset_{s}-\emptyset_{l}-j_{tot}R_{Li}-U_{lpl}$$

where $j_{0,\mathrm{lpl}}$ is the exchange current density for lithium plating, $\alpha_{c,\mathrm{lpl}}\;$ is the transfer coefficient, R  is the molar gas constant,  T  is the thermodynamic temperature, ${\;R}_{Li\;}$ is the film resistance of the plated lithium, $U_{lpl\;}$ is the equilibrium potential for lithium plating, and $\;\eta_{lpl}$ is the overpotential. Lithium plating occurs when $\eta_{lpl}<0$.

The relationship between local current density and changes in lithium plating film thickness:

(11) $$\frac{{\partial \delta }_{Li}}{\partial t}=-\frac{j_{lpl}M_{Li}}{F\rho_{Li}}$$

The relationship between film thickness and film resistance is represented as follows:

(12) $$R_{Li}=\frac{\delta_{Li}}{\sigma_{Li}}$$

The growth of the film affects the porosity of the electrode is represented as follows:

(13) $$\frac{d\varepsilon_{l}}{dt}=-a\frac{d\delta_{Li}}{dt}$$

where $\varepsilon_{l}\;$ is the porosity,$\;\delta_{Li\;}$ is the film thickness of the plated lithium, $M_{Li}\;$ is the molar mass of lithium,$\rho_{Li\;}$ is the density of lithium ${,\;\sigma }_{Li}\;$ is the electrical conductivity of lithium, and  a is the proportionality coefficient.

#### 2.1.3. Gas Generation

The construction of the gas generation model primarily considers two stages: Stage 1, where gas continuously forms inside the battery as side reactions proceed. Due to the manufacturing process, LFP/graphite batteries typically have some gaps between the aluminum casing and the cell, which are initially occupied by the produced gas. In Stage 2, after these gaps are filled, the subsequently generated gas diffuses into the cell interior, occupying the electrode pores and causing partial deactivation of active materials.

The gas generated by the battery can be quantitatively calculated using the following equation:

(14) $$\frac{\partial V_{gas}}{\partial t}=\frac{A_{0}\tilde{V}\int a_{p}j_{SEI}dL_{neg}}{F}\varphi$$

where $\;V_{gas\;}$ is the total volume of generated gas, $A_{0}$ is the area of the electrode involved in the electrochemical reaction, set to 1 $m^{2}$ in the model, $\tilde{V}\;$ is the molar volume of an ideal gas, φ is the gas generation control factor, $\;a_{p}$ is the specific surface area per unit thickness in the direction of the anode, and $L_{neg}$ is the thickness of the anode.

When the volume of the generated gas has not yet exceeded the gaps, the gas generation does not yet impact the electrochemical process.

The gap around the battery is denoted as $V_{m\arg in}$. Here, it is assumed that the total gap constitutes 5% of the volume of the electrode and separator, i.e.,  ξ is 5%:

(15) $$V_{m\arg in}=\xi A_{0}(L_{neg}+L_{sep}+L_{pos})$$

$L_{sep\;}$ is the thickness of the separator, and $\;L_{pos}\;$ is the thickness of the positive electrode.

The total volume of the pores inside the battery is denoted as $V_{pore}$.

(16) $$V_{pore}=A_{0}\left(L_{neg}\varepsilon_{l}^{neg}+L_{sep}\varepsilon_{l}^{sep}+L_{pos}\varepsilon_{l}^{pos}\right)$$

$\varepsilon_{l}^{neg},\;\varepsilon_{l}^{sep}and\;\varepsilon_{l}^{pos}$ represent the porosity of the anode, separator, and cathode, respectively.

As gas continues to accumulate and fills all the gaps, the subsequently generated gas begins to diffuse into the electrode interior. Two new variables are introduced here to characterize the impact of this process on the electrochemical reaction.

(17) $$\theta =\frac{V_{remain}}{V_{pore}}$$

(18) $$V_{remain}=V_{pore}-(V_{gas}-V_{m\arg in})$$

where θ  is the gas generation impact factor, and $V_{remain}$ is the volume of the remaining pores. When $\;V_{gas}>V_{m\arg in}$, the gas diffuses into the interior, reducing the porosity of the electrodes and separator, as well as the activity of the electrode particles. At this point, the internal parameters of the electrode are updated as follows:

(19) $$\varepsilon_{l}^{\prime }=\varepsilon_{l}\times \theta$$

(20) $$\varepsilon_{s}^{\prime }=\varepsilon_{s}\times \theta$$

$\varepsilon_{l}{\;and\;\varepsilon }_{s}$ are the initial porosity and solid-phase volume fraction of the battery, respectively, while $\varepsilon_{l}^{\prime }\;and\;\varepsilon_{s}^{\prime }\;$ are the updated current porosity and volume fraction of active material after adjustment.

In the reported aging models, the coupling relationships between the aforementioned side reactions are rarely considered. However, during actual aging processes, these side reactions interact with each other. If the coupling relationships between the side reactions are not considered, the accuracy of the model may decrease. Therefore, based on the classic P2D simulation model [19,20], we used commercial simulation software COMSOL 5.6 and incorporated some micro-level parameters obtained from cell disassembly to establish a battery mechanistic model. This model is built on NEWMAN’s electrochemical model and integrates electrochemical reaction kinetics, mass conservation, and charge conservation.

The mechanistic model we propose primarily considers two dimensions: the x-dimension along the electrode thickness and the r-dimension along the radius of the electrode particles. The parameters in the mechanism model are mainly divided into three parts. The first part consists of microstructural parameters, which refer to parameters related to the electrode structure, such as electrode thickness and porosity. The second part includes mass transfer and lithium deintercalation reaction kinetics parameters, which pertain to the kinetics of the deintercalation reaction and mass transfer processes and typically require acquisition through electrochemical experiments or reference to literature values. The final part comprises aging reaction parameters, which are kinetic parameters related to side reactions and generally require calibration based on aging experimental data.

### 2.2. Model Parameter Determination

**Figure 1:**
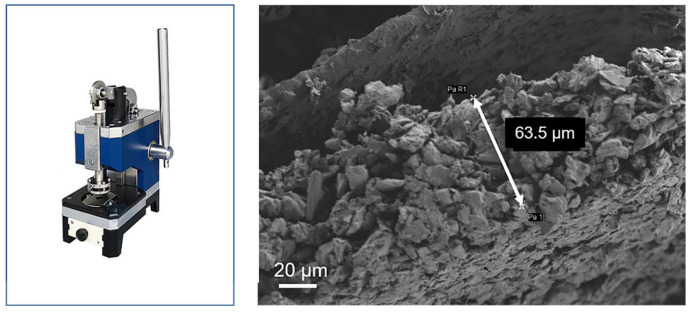
Sample stamping machine and cathode thickness SEM image.

**Figure 2:**
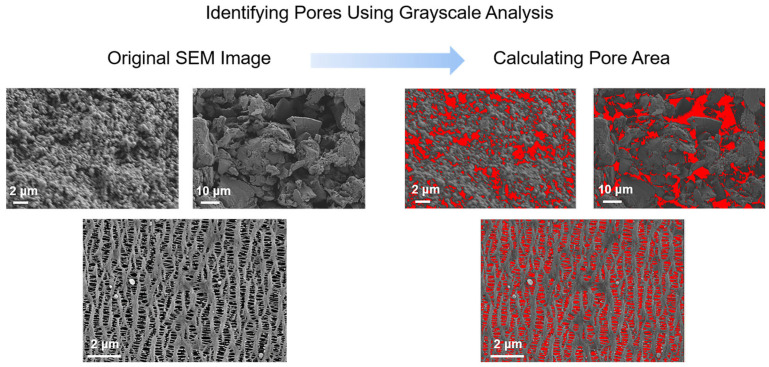
Grayscale identification for porosity measurement.

**Figure 3:**
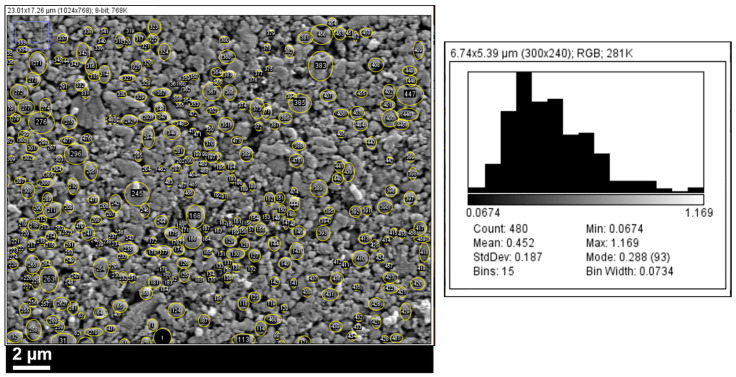
Acquisition of the anode and cathode fret radius.

By disassembling the actual cell and using a scanning electron microscope (SEM) along with post-processing software, we obtained the battery’s micro-level parameters, including the thickness of the anode, cathode, and separator; the particle radius of the anode and cathode; and the porosity of the anode, cathode, and separator, as shown in Figure 1, Figure 2 and Figure 3. The samples were all prepared according to the ASTM E3-11 metallographic specimen preparation standard. In brief, the electrode sheet was disassembled from the battery cell in an argon (Ar) protective atmosphere glovebox. It was then cleaned with dimethyl carbonate (DMC) solvent to remove residual electrolyte from the surface. Subsequently, the electrode sheet was cut to an appropriate size, fixed onto the sample stage using conductive adhesive, and finally characterized and analyzed for microscopic morphology using field emission scanning electron microscopy (FE-SEM, HITACHI Regulus8100, Tokyo, Japan). And the microscopic morphology of the samples were all imprinted in the SE2 mode under the FE-SEM.

The acquired micro-level parameters are shown in Table 2.

The specific parameter values, acquisition methods, and parameter classifications are shown in Table 3.

## 3. Validation of Mechanistic Model

The mechanistic model was constructed based on three typical internal aging mechanisms of batteries. Before simplifying the model and applying it in engineering contexts, it was essential to validate the model’s accuracy to ensure its reliability. This step ensured that the model maintained high precision and provided a solid foundation for subsequent engineering applications a 20nd system-level analyses. The charge–discharge test data of the cells were conducted in laboratory environments accredited by the China National Accreditation Service for Conformity Assessment (CNAS), strictly adhering to the ISO/IEC 17025 international standard. This ensured the accuracy, reliability, and traceability of the test data. The laboratory was equipped with advanced testing equipment and professional technical team, employing standardized testing procedures and rigorous quality control system, thereby providing robust guarantees for the scientific validity and authoritative credibility of the test results.

### 3.1. Validation of the Mechanistic Model at Different Temperatures

To verify the accuracy of the established mechanistic model, cycle tests were conducted on this battery model under specific conditions in the laboratory, comparing the differences between actual test results and model simulation results. Table 4 shows the cycling conditions of the cell, which were tested at 25 °C and 45 °C using constant power charge and discharge, with upper and lower cut-off voltages of 3.65 V and 2.5 V, respectively.

**Figure 4:**
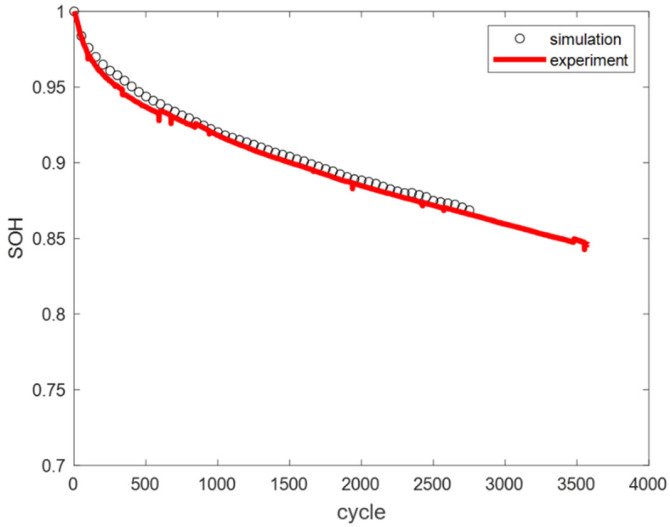
Comparison of experimental and simulated cycle capacity fade curves at 25 °C.

**Figure 5:**
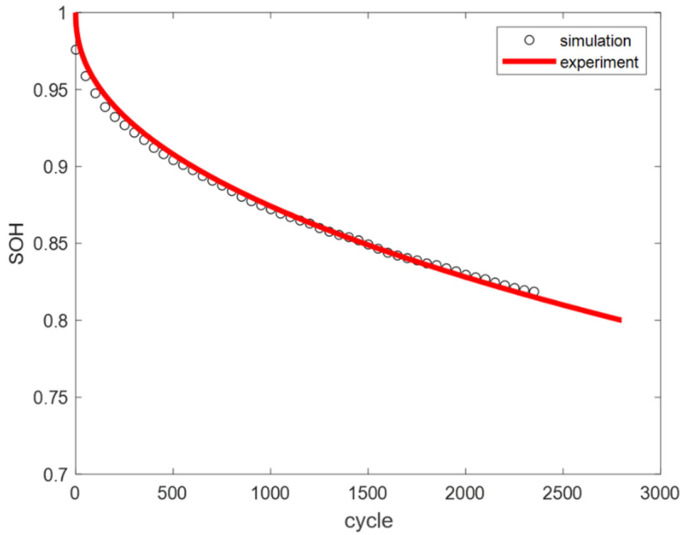
Comparison of experimental and simulated cycle capacity fade curves at 45 °C.

Based on the above conditions, the simulation results of the mechanistic model were compared with the actual test data. At 25 °C, the error between the simulation results and experimental data was 0.43%, as shown in Figure 4. At 45 °C, the error was 0.35%, as shown in Figure 5. The average model error at both temperatures was less than 1%.

### 3.2. Validation of Mechanistic Model at Different Depths of Discharge (DODs)

Based on the operating temperature and DOD range of batteries in actual field stations, corresponding cycling tests were conducted in the laboratory to validate the accuracy of the model simulation results at different DOD levels.

**Figure 6:**
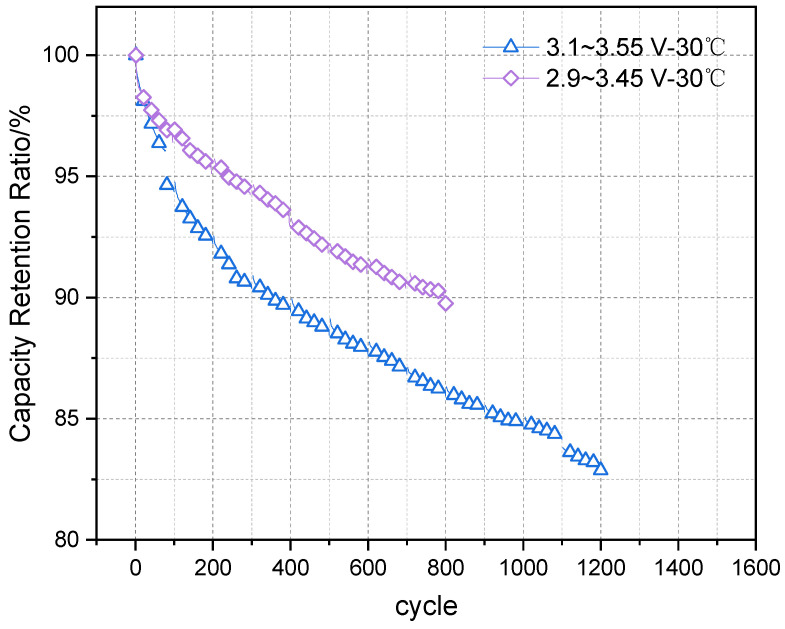

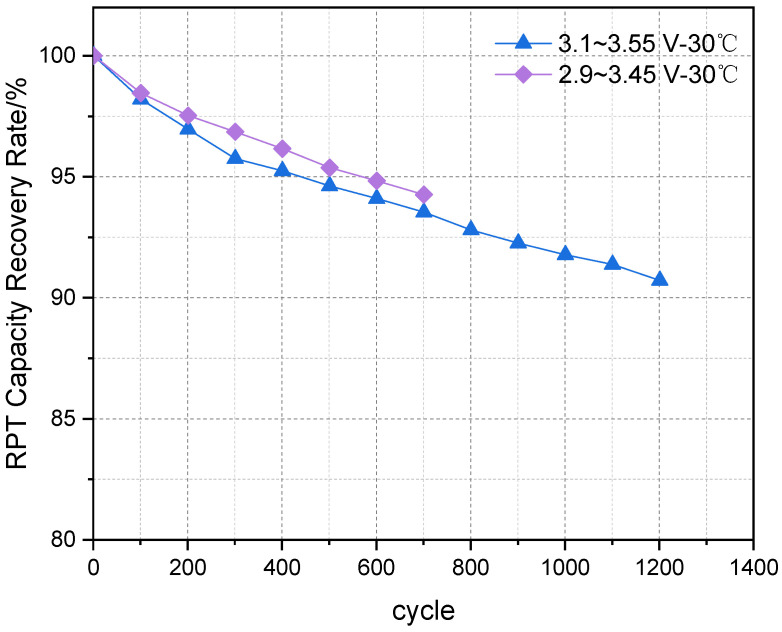
Measured cycle data under actual operating conditions.

Figure 6 shows the measured data of the cells in two different cycling modes set based on actual operating conditions. At 30 °C and 0.5P, the voltage cut-off ranges were set to 3.10 V–3.55 V and 2.90 V–3.45 V, both with a DOD of 90%. The test results indicate that the battery’s capacity retention rate decays more rapidly in the 3.10 V–3.55 V range, but the capacity fade under standard conditions (25 °C, 0.5P, 100% DOD) showed similar results in both operating modes.

**Figure 7:**
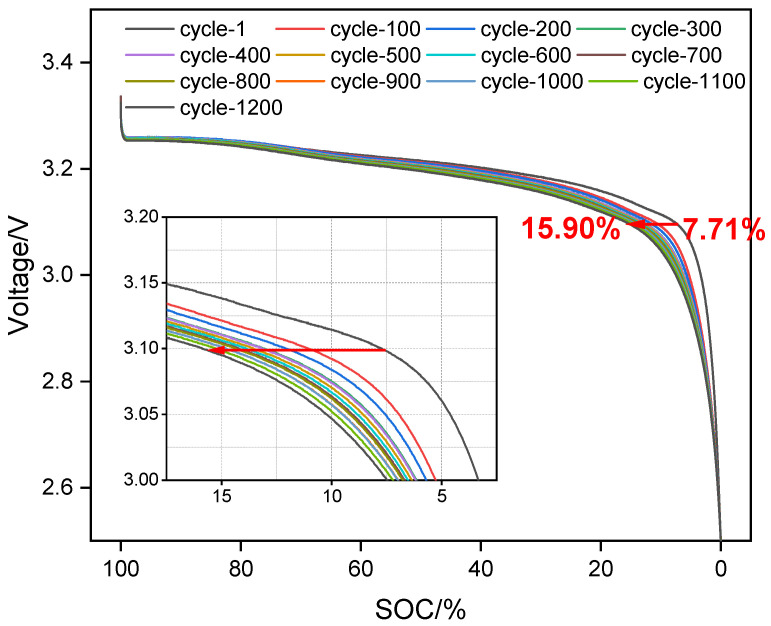

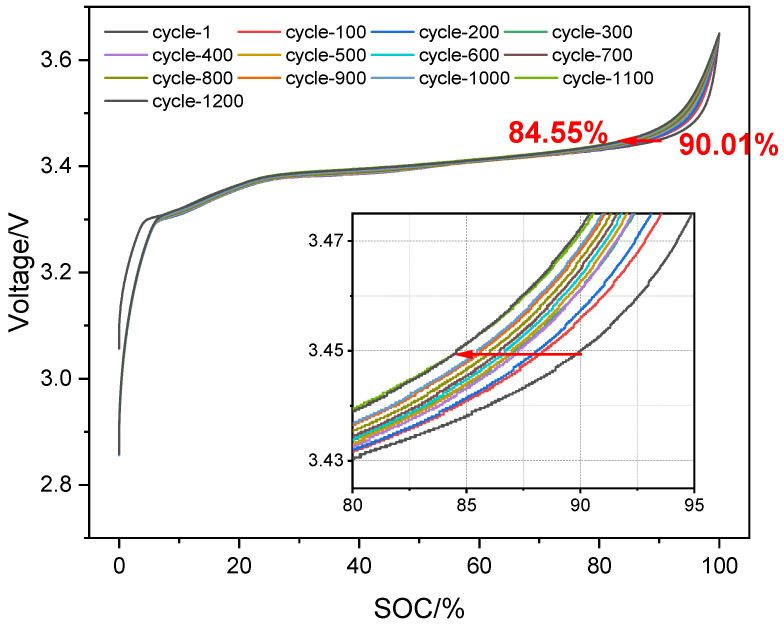
Analysis of measured charge and discharge curves for battery aging.

Based on the above test results, a detailed analysis of the charge and discharge characteristic curves during cycling (as shown in Figure 7) reveals that these curves changed as the battery aged. When discharging to 3.10 V or charging to 3.45 V, the voltage remained relatively stable, causing the actual DOD within the corresponding voltage range to decrease. This ultimately resulted in the apparent capacity retention rate of the battery declining faster than the actual RPT capacity recovery rate.

**Figure 8:**
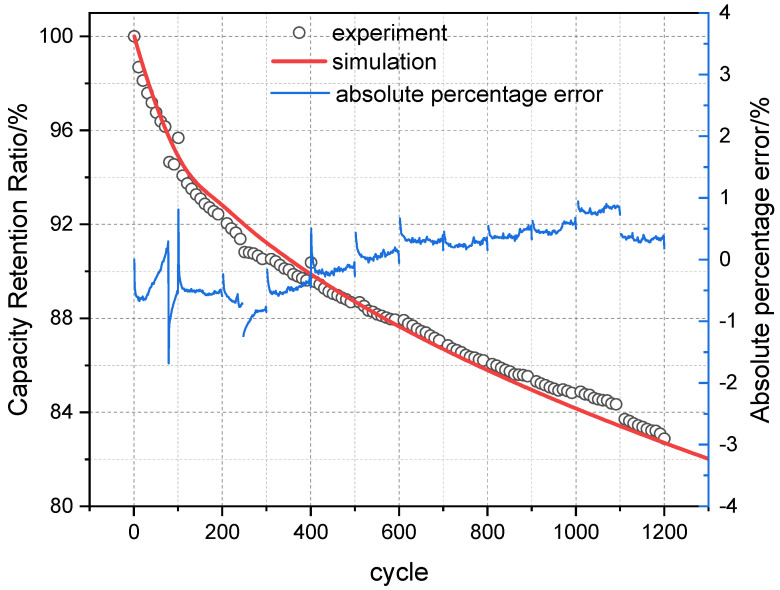
Comparison and validation of measured cycle and simulation results at 30 °C, 0.5P, and 3.10 V–3.55 V.

**Figure 9:**
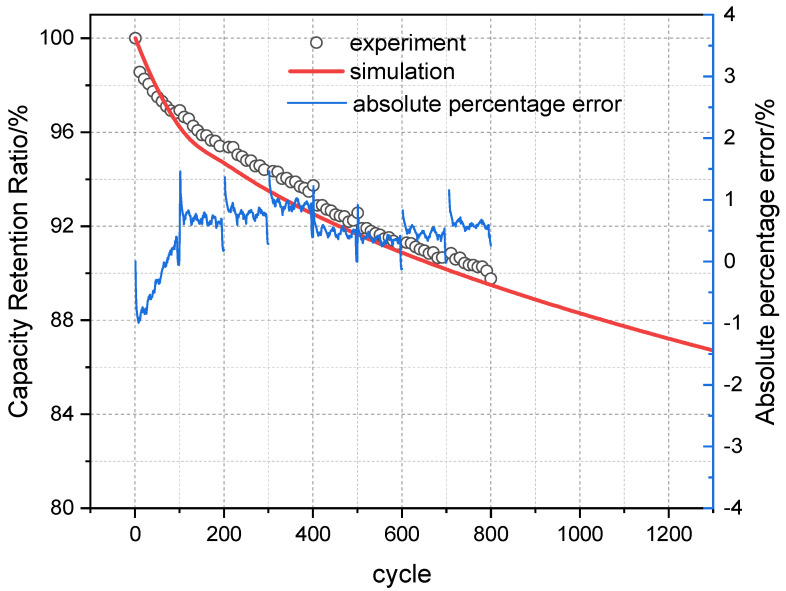
Comparison and validation of measured cycle and simulation results at 30 °C, 0.5P, and 2.90 V–3.45 V.

Based on the above conditions, simulation calculations were performed. The results are shown in Figure 8 and Figure 9. The average error between the measured data and simulation results for the two different conditions was within ±1%, indicating good simulation accuracy.

## 4. Simplification of the Mechanistic Model into the Engineering Model

Due to the lengthy computation time required for the mechanistic model, a single aging condition calculation typically takes several to tens of hours, making it challenging to apply this model directly for the rapid prediction and construction of energy storage system-level model. The mechanistic model is capable of handling complicated operating conditions including different variations in T, DOD, etc. However, in typical onsite application, the operating conditions are generally limited and regularized. Therefore, based on the mechanistic model, representative conditions were calculated to obtain internal aging information of the battery under different aging scenarios, enabling the development of a simplified engineering model.

### 4.1. Assumptions for Simplifying the Mechanistic Model

The foundation of the engineering model was to simplify the mechanistic model of the battery into two types of factors: LAM (loss of active material) and LLI (loss of lithium inventory). In different aging modes, the lithium intercalation range between the anode and cathode changes, ultimately leading to different battery capacity. First, certain assumptions need to be made regarding how LLI and LAM affect the relationship of lithium intercalation between the anode and cathode. Based on the characteristics of lithium ion batteries, the assumptions for the engineering model are as follows:

(1) Since the anode capacity is greater than the cathode capacity, the cathode capacity is used as a reference for normalization;
(2) LLI occurs during the charging process and only at the anode;
(3) LAM is caused by gas generation, so the LAM is the same for both the anode and cathode.

**Figure 10:**
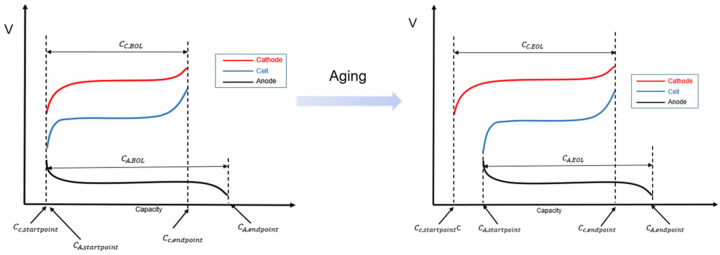
Simplified battery capacity model.

Based on these assumptions, the battery capacity model can be derived, as shown in Figure 10.

(21) $$C_{A,EOL}=C_{A,BOL}\cdot \left(1-{LAM}_{A}\right)$$

(22) $$C_{C,EOL}=C_{C,BOL}\cdot \left(1-{LAM}_{C}\right)$$

(23) $$C_{A,startpoint}=LLI\cdot C_{C,BOL}+0.5{LAM}_{A}\cdot C_{A,BOL}$$

(24) $$C_{C,start\;point}=0.5{LAM}_{C}\cdot C_{C,BOL}$$

(25) $$C_{A,endpoint}=C_{A,BOL}+LLI\cdot C_{C,BOL}-0.5{LAM}_{A}\cdot C_{A,BOL}$$

(26) $${\;C}_{C,endpoint}=C_{C,BOL}-0.5{LAM}_{C}\cdot C_{C,BOL}$$

(27) $$C_{Cell}=\min\left(C_{A,endpoint},C_{C,endpoint}\right)-\max(C_{A,startpoint},C_{C,startpoint})$$

where $C_{A,BOL}\;$ is the initial anode capacity, $C_{C,BOL}$ is the initial cathode capacity, $C_{A,EOL}\;$ is the anode capacity after aging, $\;C_{C,EOL}$ is the cathode capacity after aging, $C_{A,startpoint}\;$ is the position when the anode SOC = 0%, $\;C_{C,start\;point}\;$ is the position when the cathode SOC = 0%,${\;C}_{A,endpoint}\;$ is the position when the anode SOC = 100%, ${\;C}_{C,endpoint\;}$ is the position when the cathode SOC = 100%, ${\;C}_{Cell}\;$ is the normalized battery capacity, ${LAM}_{A\;}$ is the loss of active material in the anode, and $\;{LAM}_{C}$ is the loss of active material in the cathode.

The simplified battery capacity model above includes two core parameters: LLI and LAM. The variation in LLI is caused by SEI film growth and lithium plating in the mechanistic model, allowing for capacity loss due to SEI film growth and lithium plating side reactions to be calculated through model simulation.

(28) $$Q_{LLI}=\int_{0}^{L_{neg}}\int_{t=0}^{t_{1}}j_{SEI}(x,t)dtdx+\int_{0}^{L_{neg}}\int_{t=0}^{t_{1}}j_{lpl}(x,t)dtdx$$

$Q_{LLI}\;$ is the capacity loss caused by LLI,  x  represents the direction of thickness, and $\;j_{lpl}\;$ is the current density of the lithium plating reaction.

Based on the assumptions of the simplified mechanism model, LAM is primarily caused by gas generation and cannot be directly calculated through the model. However, since the overall capacity loss of the battery is mainly due to LLI and LAM, LAM can be indirectly obtained by calculating the difference between the total capacity loss of the battery and the capacity loss caused by LLI. Capacity calibration tests at 1/25C were conducted on batteries with different cycle counts to obtain their thermodynamic capacity *Q*_aging_. The capacity loss due to LAM can be calculated as follows:

(29) $$Q_{LAM}=Q_{aging}-Q_{LLI}$$

### 4.2. Data Fitting Results of LLI and LAM

The establishment of the engineering model requires determining the trend of changes in LLI and LAM within the battery, which is obtained through calculations from the mechanism model. In the model, LLI and LAM are defined as follows: LLI is determined by calculating the SEI film growth and lithium plating amount on the particle surface, while LAM is obtained by the difference between the total thermodynamic capacity and LLI. First, the mechanism model was used to calculate the aging decay curves of the battery at 0.5P and 25 °C/35 °C/45 °C (where the calculation results of the mechanistic model at 25 °C and 45 °C were experimentally validated). The LLI values at 25 °C, 35 °C, and 45 °C were then obtained. Using the difference between the thermodynamic capacity of the battery measured at these three temperatures and the calculated LLI, LAM was determined. Finally, the resulting LLI and LAM variation curves were fitted with functions to derive a simplified engineering model.

**Figure 11:**
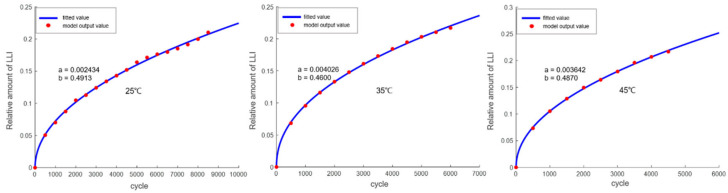
Fitting of the LLI variation trend output by the mechanism model.

The trend of LLI variation is shown in Figure 11, and the fitted function is as follows:

(30) $$LLI=a\times x^{b}$$

**Figure 12:**
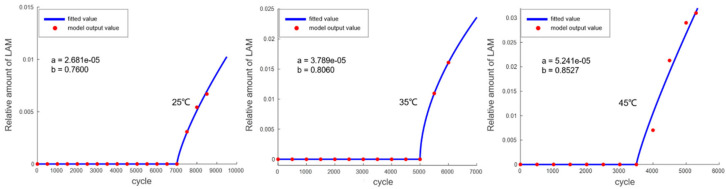
Fitting of the LAM variation trend output by the mechanism model.

The trend of LAM variation is shown in Figure 12, and the fitted function is as follows (where *G* represents the onset point of gas generation):

(31) LAM=0 (x≤G)

(32) $$LAM=a\times {\left(x-G\right)}^{b}\;(x>G)$$

**Figure 13:**
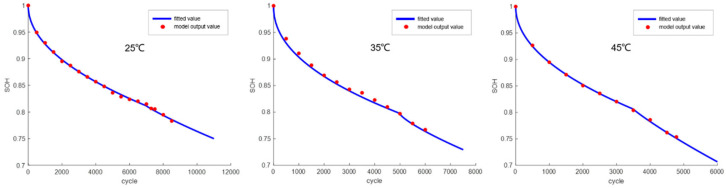
Capacity decay curves under different temperature conditions.

Based on the LLI and LAM fitting functions established through the above analysis, the calculated SOH decay values of the battery under three temperature conditions were compared with the values calculated by the mechanism model, as shown in Figure 13. The results show a close match between the two, indicating that the determination of these fitting functions is effective.

Using the engineering model described above, assumptions can be made about the variation in coefficients in the fitting formula with operating conditions (such as temperature), thereby obtaining the battery decay patterns under different conditions and generating aging datasets for constructing a data-driven model. Here, temperature was used as an example to illustrate the generation process of the aging dataset required for the data-driven model.

The relationship between the parameters in the fitting formula and temperature is described by the Arrhenius equation:

(33) $$ln\left(k\right)=\frac{E_{a}}{RT}+ln\left(A\right)$$

where k is the reaction rate constant,  A  is the pre-exponential factor, ${\;E}_{a}$ is the activation energy, T  is the absolute temperature, and R is the molar gas constant.

**Figure 14:**
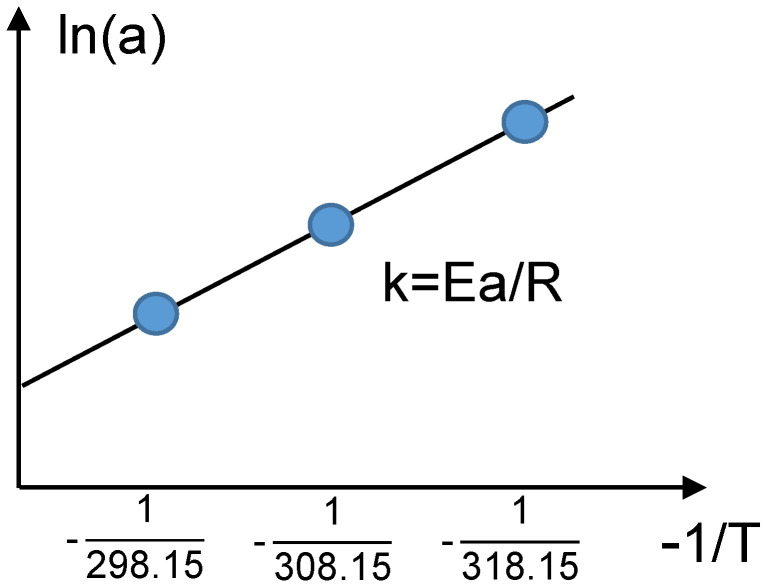
Arrhenius coefficient calculation.

As shown in Figure 14, the coefficients of the LLI and LAM variation functions at 25 °C, 35 °C, and 45 °C were fitted using the Arrhenius equation to obtain the reaction coefficients a and b at different temperatures T.

**Figure 15:**
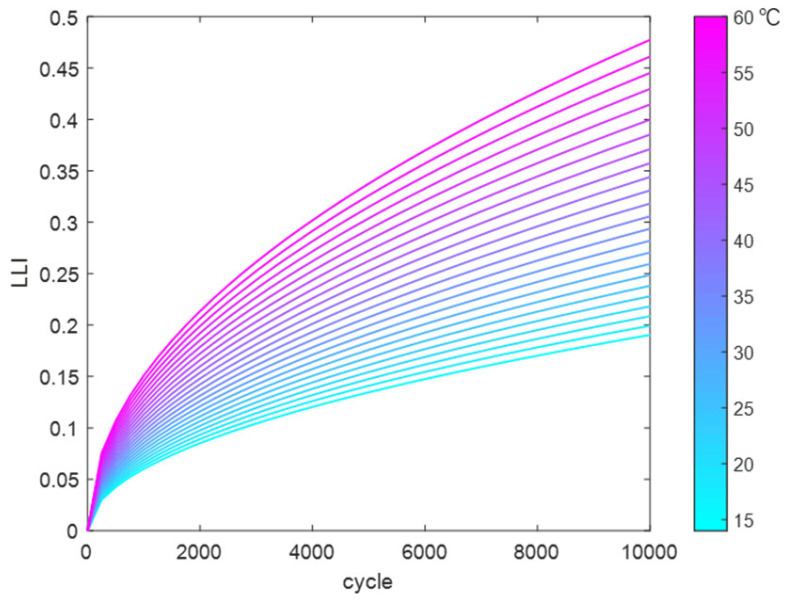
LLI variation dataset at different temperatures.

Using the above method, the reaction coefficients in the LLI fitting function were fitted and solved to obtain a temperature-dependent fitting function. This was used to calculate the trend of LLI variation with cycle count at different temperatures (14 °C to 60 °C), as shown in Figure 15.

**Figure 16:**
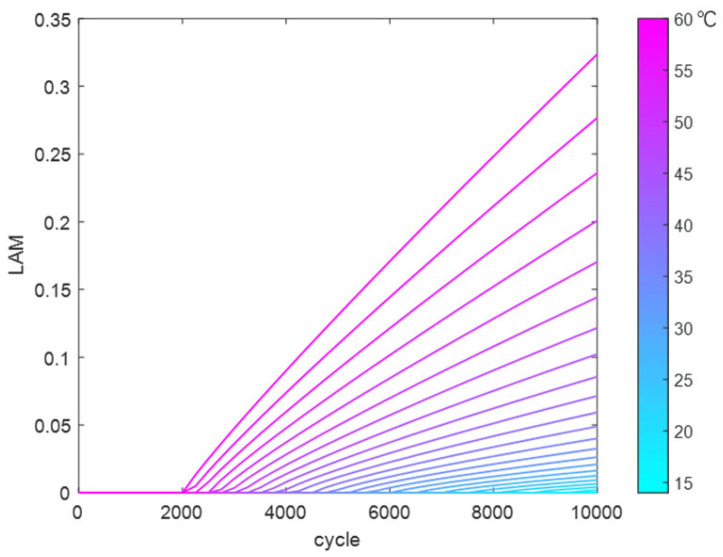
LAM variation dataset at different temperatures.

Similarly, the reaction coefficients in the LAM fitting function were fitted and solved to obtain a temperature-dependent fitting function. This was used to calculate the trend of LAM variation with cycle count at different temperatures (14 °C to 60 °C), as shown in Figure 16.

**Figure 17:**
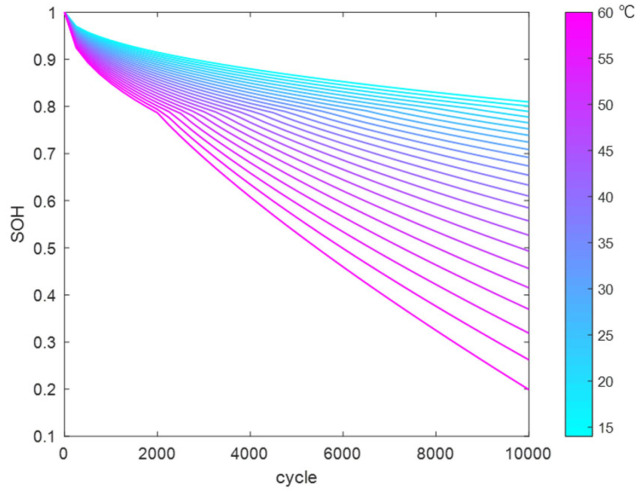
SOH variation at different temperatures.

By substituting the LAM and LLI datasets into the model, the battery SOH variation curves under SEI film growth and gas generation mechanisms at different temperatures can be obtained, as shown in Figure 17.

The engineering model established using the above method can calculate the capacity decay curve under different temperature conditions. Compared to the mechanism model, this model has only a few parameters, which significantly reduces computation time while ensuring model accuracy, making it suitable for rapid SOH calculations in practical engineering projects. The error propagation in the aging model primarily stems from the following three aspects: parameter identification, simplification of nonlinear dynamic characteristics, and limitations of data samples. To effectively reduce error accumulation, future research will integrate the interpretability of mechanistic models with the adaptability of data-driven models to conduct multi-model fusion studies. Additionally, based on identified key factors affecting lifespan degradation (such as temperature, DOD, and C-rate), more cross-orthogonal experiments will be designed to dynamically calibrate model parameters, thereby continuously enhancing the robustness and predictive accuracy of the models.

### 4.3. Validation of Engineering Model Under Onsite Operating Conditions

**Figure 18:**
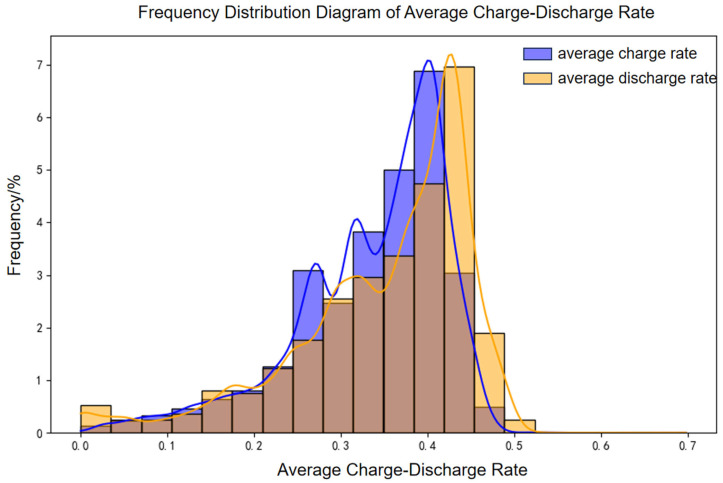
Frequency distribution diagram of average charge–discharge rates.

**Figure 19:**
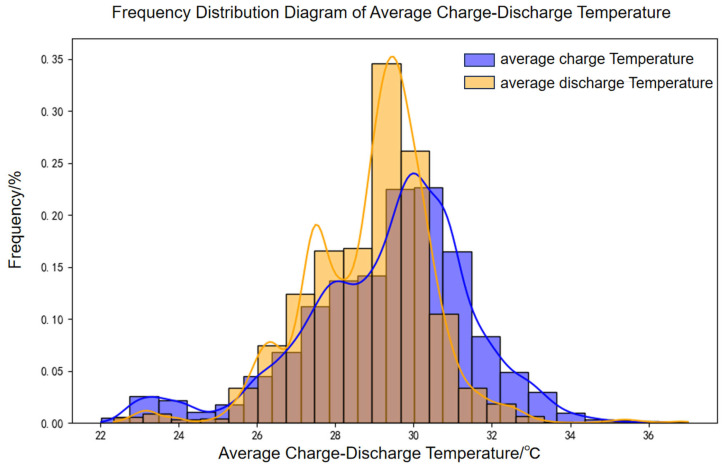
Frequency distribution diagram of average charge–discharge temperatures.

A 100 MW/200 MWh energy storage power station was selected to evaluate battery aging under real-world operating conditions using an established engineering model. The accuracy of the model was subsequently validated through measured data. Operational data from March 2022 to December 2024 were analyzed, with the distributions of charge–discharge rates and temperatures depicted in Figure 18 and Figure 19, respectively.

**Figure 20:**
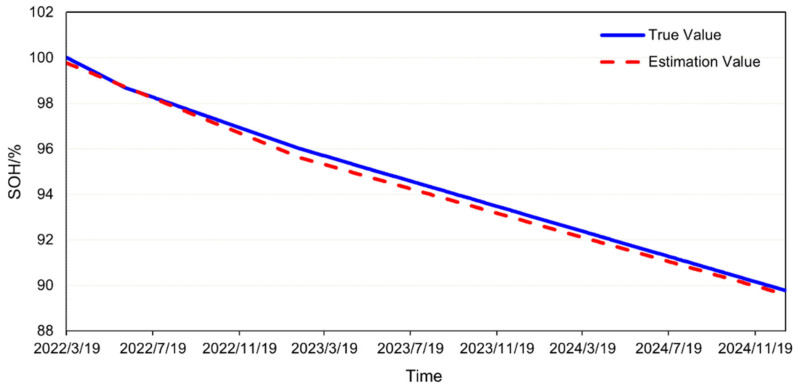
Validation result of the engineering model.

Under actual operating conditions, the measured SOH of the battery and the SOH calculated by the engineering model are compared in Figure 20. As of 31 December 2024, the measured SOH of the battery was 89.78%, while the engineering model predicted an SOH of 89.61%, resulting in an error of −0.17%. These findings demonstrate that the engineering model exhibited high accuracy and was capable of reliably predicting battery aging under complex operational conditions. Under identical hardware configurations (Intel(R) Xeon(R) Platinum 8260 CPU @ 2.40 GHz 2.39 GHz, 1024 GB RAM, 8 × 3.84 TB SATA SSD), the computational time required for estimating the SOH of the battery using a mechanistic model was 8.12 h, whereas the engineering model accomplished the same task in a mere 10 s. This significant disparity in computational efficiency underscores the advantage of the engineering model, which not only expedites the SOH estimation process, but also facilitates the scalability of the model to system-level applications, thereby enhancing its potential for large-scale engineering deployment.

## 5. Conclusions

The mechanistic model in this study addresses the limitations of empirical LFP/graphite cell aging model in adequately accounting for side reactions on the cathode side. Based on the P2D model, it specifically models the three aging mechanisms unique to the LFP/Graphite system: SEI film growth, lithium plating, and gas generation. Under different temperature and DOD conditions, the average error between the simulation results of this model and actual measurements was within ±1%, demonstrating high accuracy and adaptability.

Additionally, to facilitate engineering applications of the model, a simplified engineering model was developed based on the matching relationship between the anode and cathode, consisting of two core parameters: LLI and LAM. This engineering model, grounded in the cell’s aging mechanisms, calculates and outputs LLI and LAM values under various conditions, followed by regression fitting and simplification. This approach significantly reduces the model parameters and computational load, making it suitable for large-scale engineering applications, and also provides a foundation for building model for energy storage systems. The validation of the engineering model within a 100 MW/200 MWh energy storage project revealed a minimal deviation of −0.17% from the actual State of Health (SOH) when the battery’s SOH had degraded to 89.78%. Compared to the mechanistic model, the computational time under the same hardware configuration was reduced from 8.12 h to 10 s. At the technology transformation level, it is expected to be divided into the following aspects: (1) industry adaptation approach: simplify the computing framework and align it with the industry-standard hardware and software ecosystem; (2) deployment case studies: work with MWh-class energy storage plants to pilot, and continuously validate to optimize model accuracy; (3) scalability analysis: evaluate the cost of cloud-based deployments and the analytics latency of million-battery databases.

In subsequent work, efforts will focus on developing a comprehensive energy storage system model based on the engineering model, which incorporates multiple battery cells connected in series and parallel. This model will be integrated with an auxiliary power consumption model of the thermal management system to establish a coupled energy efficiency–lifetime model. The aim is to accurately calculate and optimize the actual discharge capacity of large-scale energy storage systems, thereby enhancing the overall economic benefits of energy storage stations. This approach will provide a robust framework for improving the operational efficiency and profitability of energy storage facilities.

## Figures and Tables

**Table 1:** Operating conditions exacerbating the side reactions.

Side Reaction	Subset	Temperature	Voltage or SOC	Current
Decomposition and Growth of SEI		High Temperature	High Voltage	Not Applicable (N/A)
Lithium Plating		Low Temperature	High Voltage	High C-rate
Particle Fracture	Graphite	Low Temperature	Multiple SOC Ranges	High C-rate
	NCM	Low Temperature	High SOC	High C-rate
	LFP	Low Temperature	High SOC	High C-rate
Structural Changes and Decomposition of the Cathode	Phase Transition	High Temperature	High Voltage and High SOC	High C-rate
	Electrochemical Decomposition (Oxidation of Crystalline Oxygen	High Temperature	High Voltage	High C-rate
	Cation Mixing	High Temperature	High Voltage	N/A
	Acid Corrosion	High Temperature	N/A	N/A
	Reaction with Electrolyte	High Temperature	High Voltage	N/A
Gas Generation		High Temperature	High SOC	N/A
Graphite Exfoliation		N/A	N/A	N/A

**Table 2:** Micro-level parameters of the battery.

Name	Parameter	Value	Name	Parameter	Value
Cathode Thickness	L_pos	96 µm	Cathode Solid-Phase Volume Fraction	epss_pos	0.743
Anode Thickness	L_neg	66 µm	Cathode Porosity	epsl_pos	0.257
Separator Thickness	L_sep	20 µm	Separator Porosity	epsl_sep	0.269
Cathode Particle Radius	rp_pos	0.275 µm	Anode Solid-Phase Volume Fraction	epss_neg	0.813
Anode Particle Radius	rp_neg	5.72 µm	Anode Porosity	epsl_neg	0.187

**Table 3:** Model parameter values.

Parameter Type	Parameter	Cope			Acquisition Method
		Anode	Separator	Cathode	
Structural Parameter	L ** (μm ** **)**	66	20	96	Microscopic characterization experiments
	$\varepsilon_{l}$	0.187	0.5	0.332	
	$\varepsilon_{s}$	0.813	/	0.618	
	$R_{p}\;$ ** (μm ** **)**	5.72 m	/	0.275	
Mass Transfer and Lithium Deintercalation Kinetics Parameters	$x_{100\%}$	0.811	/	0.035	Obtained by fitting the anode, cathode and full cell OCV curves
	$x_{0\%}$	0.0132	/	0.74	
	$k_{1}(m\cdot s^{-1})$	$1.3\times 10^{-10}$	/	$4.2\times 10^{-10}$	From literature values (COMSOL built-in)
	$D_{s}(m\cdot s^{-1})$	$3.9\times 10^{-14}$	/	$1.25\times 10^{-15}$	
	$D_{e}(m\cdot s^{-1})$	The order of magnitude is generally 1 S/m; specific parameters refer to the COMSOL model.			
Aging-Related Parameters	$k_{2}(m\cdot s^{-1})$	$1\times 10^{-12}$	/	/	From literature values
	$k_{SEI}(S\cdot m^{-1})$	$5\times 10^{-6}$	/	/	Obtained by comparison with cycle aging experiments
	$V_{EC}^{M}\;\left(m^{3}/mol\right)$	$9.6\times 10^{-5}$			
	$D_{EC}\;\left(m^{2}/s\right)$	$6\times 10^{-21}$			
	$M_{SEI}\;\left(kg/m^{3}\right)$	0.162			
	θ	1.95			
	α	0.002			
	$C_{EC}^{0}(mol/m^{3})$	4541			From literature values
	$\rho_{SEI}\;\left(kg/m^{3}\right)$	1690			From literature values

**Table 4:** Charge and discharge parameters for model simulation.

Parameter	Illustration
Anode Material/Cathode Material	Graphite/LiFePO_4_
Rated Capacity	280 Ah
Rated Voltage	3.2 V
Rated Power	0.5 × 280 × 3.2 V
Charge/Discharge Cut-off Voltage	3.65 V/2.5 V
Temperature	25 °C/45 °C

## Data Availability

The original contributions presented in this study are included in the article. Further inquiries can be directed to the corresponding author.
